# Estimating the parasitaemia of *Plasmodium falciparum*: experience from a national EQA scheme

**DOI:** 10.1186/1475-2875-12-428

**Published:** 2013-11-22

**Authors:** Monika Manser, Catherine Olufsen, Nick Andrews, Peter L Chiodini

**Affiliations:** 1 UKNEQAS Parasitology, Department of Clinical Parasitology, Hospital for Tropical Diseases, Mortimer Market, Capper Street, London WC1E 6JB, UK; 2 Statistics department, Public Health England, 61 Colindale Avenue, London NW9 5HT, UK; 3 Malaria Reference Laboratory, London School of Hygiene and Tropical Medicine, Keppel Street, London WC1E 7HT, UK

## Abstract

**Background:**

To examine performance of the identification and estimation of percentage parasitaemia of *Plasmodium falciparum* in stained blood films distributed in the UK National External Quality Assessment Scheme (UKNEQAS) Blood Parasitology Scheme.

**Methods:**

Analysis of performance for the diagnosis and estimation of the percentage parasitaemia of *P. falciparum* in Giemsa-stained thin blood films was made over a 15-year period to look for trends in performance.

**Results:**

An average of 25% of participants failed to estimate the percentage parasitaemia, 17% overestimated and 8% underestimated, whilst 5% misidentified the malaria species present.

**Conclusions:**

Although the results achieved by participants for other blood parasites have shown an overall improvement, the level of performance for estimation of the parasitaemia of *P. falciparum* remains unchanged over 15 years. Possible reasons include incorrect calculation, not examining the correct part of the film and not examining an adequate number of microscope fields.

## Background

In the UK, there are between 1,500 and 2,000 imported cases of malaria reported each year [1]. of which about 10 percent are seen at the Hospital for Tropical Diseases, with the remainder distributed throughout the UK. Approximately 12,000 cases are reported annually in Europe [2]. Accurate laboratory diagnosis is essential, particularly to detect infections with the potentially fatal *Plasmodium falciparum*. The primary method of diagnosis of malaria in most clinical laboratories is still by the microscopic examination of thick and thin blood films.

The UKNEQAS Blood Parasitology Scheme was established in 1986 in order to improve the microscopic diagnosis of blood parasites by the examination of blood films from patients with parasitic infections. The scheme was also designed to provide teaching material illustrating unusual or uncommon parasites and targeting areas where a particularly poor performance was noted.

Participants in the scheme are required to identify blood parasites present in either thick or thin blood films using light microscopy. They are also required to estimate the percentage parasitaemia if *P. falciparum* is present since this parameter has implications for prognosis and the treatment regimen employed.

This study analysed the performance of participants in the diagnosis and estimation of the percentage parasitaemia of *P. falciparum* sent in 17 distributions over a 15-year period.

## Methods

The Blood Parasitology Scheme consists of eight distributions per annum containing one or two specimens dispatched to 284 laboratories (57 UK and 227 overseas). Participation in the scheme is summarized in Table 1.

**Table 1:** Participation in the scheme

**Country**	**No of participants**
**Austria**	9
**Belgium**	3
**Denmark**	14
**Finland**	4
**France**	1
**Germany**	3
**Greece**	4
**China (Hong Kong)**	2
**Iceland**	1
**Ireland**	9
**Israel**	6
**Italy**	78
**Malawi**	1
**Netherlands**	18
**Norway**	7
**Portugal**	10
**Saudi Arabia**	1
**Slovenia**	1
**South Africa**	3
**Sweden**	25
**Switzerland**	16
**Uganda**	1
**UK**	57
**Total**	**284**

Blood samples are acquired mainly from the Department of Clinical Parasitology, Hospital for Tropical Diseases, but may also be obtained from specialist institutes. The specimens distributed are thin and/or thick blood films and may include any species of malaria or microfilaria or African trypanosomes. Leishmania species in tissue dabs are also despatched. Blood films are made by applying 3.5 microlitres of blood using an electronic AutoRep E Repeating Dispenser to each of 350 microscope slides as soon as the specimen is received. The blood specimen is re-mixed thoroughly after every 10 slides to ensure homogeneity of the samples. Thin blood films are fixed in methanol and stained with the appropriate stain immediately after slide production. Thick films are stained using Field’s stain [3] method and then a coverslip is applied using DPX mounting media. Prior to distribution, malaria species are confirmed by the polymerase chain reaction. A number of slides from each batch are also checked to ensure that the morphology of the parasites has been maintained during slide production and that there is uniformity in content between slides. Participants are requested to examine the blood film microscopically for parasites and to identify the species present with the help of clinical details provided. At least one distribution per year consists of a Giemsa-stained thin blood film containing *P. falciparum*. Participants are expected to identify the species and to estimate the percentage parasitaemia by expressing the number of infected cells as a percentage of the red blood cells [4]. The reference mean percentage parasitaemia is estimated by the staff at the Hospital for Tropical Diseases by calculating the parasitaemia from 20 slides and determining the mean. The participants’ mean percentage parasitaemia is calculated by taking the parasitaemia reported by all participants and calculating the mean. From those results, the standard deviation is determined. Results >3 standard deviations from the mean are then omitted and a new mean and standard deviation calculated. This new mean is referred to as the participants’ mean. Results are collated and analysed and a full report is provided to all participants, which includes an analysis of their individual performance and a breakdown of participants’ results. Participants are awarded maximum points if they correctly identify the parasite present and report the parasitaemia within 1 standard deviation from the mean. Points are deducted when the parasitaemia reported is greater than 1 standard deviation from the mean, when an incorrect malaria parasite is reported and when the parasitaemia is not specified.

Education is an important component of UKNEQAS so the report includes comments on participants’ performance. A teaching sheet is provided if poor performance is noted or an unusual parasite is distributed.

## Results

Between 4% and 54% (mean = 23% and median = 21.5%) of participants failed to estimate the percentage parasitaemia; between 9% and 35% (mean = 18% and median =15%) overestimated the percentage parasitaemia; between 0% and 25% (mean = 6%, median =4%) underestimated the percentage parasitaemia and between 0 and 36% (mean = 5% and median = 2.4) misidentified the malaria species present (Table 2).

**Table 2:** **Analysis of the parasitaemia results of 17 specimens containing *Plasmodium falciparum***

**No**	**Reference mean parasitaemia**	**Participants mean parasitaemia**	**Standard deviation**	**No. and % of participants reporting parasitaemia > reference mean**	**% over estimating parasitaemia**	**% under estimating parasitaemia**	**% not specifying parasitaemia**	**Incorrect *Plasmodium *** **species reported**
**1**	0.1%	0.5%	0.4	136 (62%)	12%	0%	22 (10%)	*P. vivax* 16/217 (7%)
								*P. ovale* 10/217 (5%)
**2**	0.25%	0.4%	0.3	105 (49%)	17%	0%	50 (19%)	*P. vivax* 44 (17%)
								*P. ovale* 37 (14%)
								*P. malariae* 13 (5%)
**3**	1.1%	1.4%	0.57	116 (42%)	18%	5%	NA*	None
**4**	1.4%	1.5%	0.69	85 (43%)	14%	2%	74 (27%)	*P. vivax* 1/198 (0.3%)
								*P. malariae* 1 (0.3%)
**5**	1.8%	2.3%	0.8	123 (59%)	15%	10%	58 (22%)	*P. vivax* 15/266 (6%)
								*P. ovale* 5
								(2 %)
								*P. malariae* 3 (1%)
**6**	2.5%	2.8%	1.3	75 (44%)	11%	8%	NA*	*P. vivax* 5/170 (3%)
								*P. ovale* 2/170 (1%)
**7**	2.5%	3%	1.3	127 (62%)	14%	3%	66 (24%)	*P. vivax* 10/271 (4%)
								*P. ovale* 1 (0.4%)
								*P. malariae* 2 (0.7%)
**8**	2.5%	3%	1	138 (66%)	19%	2%	49 (19%)	*P. vivax* 7 (3%)
**9**	3%	3.5%	1.1	68 (66%)	12%	4%	170 (54%)	*P. malariae* 1/236 (0.4%)
**10**	5%	5.8%	2	91 (51%)	13%	10%	66 (27%)	*P. vivax* 3/243 (1%)
								*P. ovale* 4 (1.6%)
								*P. malariae* 20 (8%)
**11**	7.5%	11.8%	4.4	98 (35%)	9%	4%	NA*	*P. ovale* 1/115 (1%)
**12**	9%	9.2%	2.5	92 (45%)	20%	19%	53 (21%)	None
**13**	10.5%	11.5%	3.4	96 (54%)	30%	3%	79 (31%)	*P. ovale* 1/256 (0.4%)
								*P. malariae* 1 (0.4%)
**14**	16%	18%	4.5	130 (57%)	35%	3%	9 (4%)	*P. vivax* 2/226 (1%)
**15**	20%	24.2%	4.6	99 (46%)	33%	4%	NA*	None
**16**	20%	20.8%	6.7	120 (40%)	25%	0	NA*	*P. vivax* 1/300 (0.3%)
**17**	30%	32%	10	80(39%)	10%	25%	52 (21%)	*P. vivax* 1/253 (0.4%)
								(*Babesia* sp. 4 (2%))

NA* Not applicable (see text below). Specimen 8 also contained trophozoites of *P. ovale* and specimen 14 also contained trophozoites of *P.vivax.*

The number of participants failing to estimate the percentage parasitaemia was not applicable to five specimens. This is because at the start of the scheme, participants were asked to calculate the percentage parasitaemia of *P. falciparum* in the given specimen. In 1994, it was decided that because UK NEQAS specimens should mimic clinical requests as much as possible, the request for all blood parasites should be changed to “Examine for parasites” and participants were then expected to calculate the percentage parasitaemia if *P. falciparum* infection was diagnosed.

**Figure 1:**
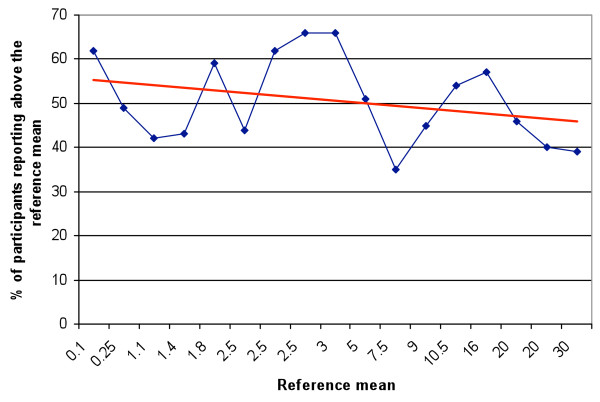
Percentage of participants reporting a parasitaemia above the reference mean (Blue line: reference mean).

**Figure 2:**
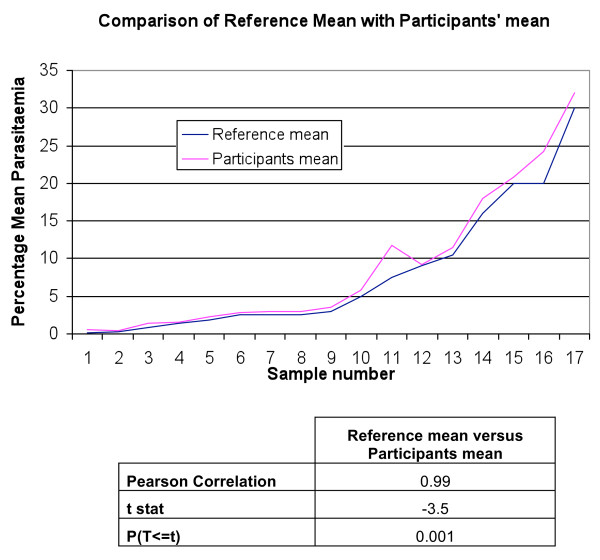
Comparison of reference mean with participants's mean (Blue line: Reference mean, Red line: participant's mean).

Statistical analyses showed that:

No significant difference was found in the percentage of participants overestimating the parasitaemia when the reference mean was high compared with when it was low. (P = 0.27, regression statistics) (Figure 1). Though always greater, the participants’ mean was in good agreement with the reference mean (P(T < =t) 0.001 (Figure 2).

**Figure 3:**
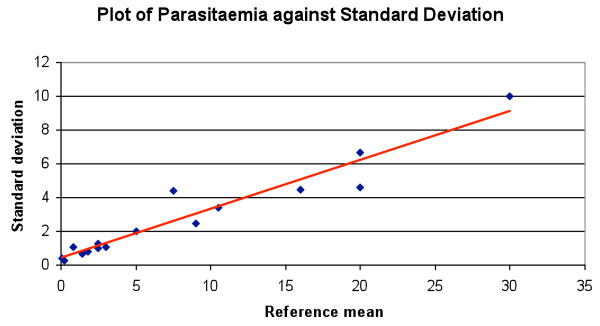
Plot of reference mean parasitaemia against standard deviation (Blue: value, Red line: predicted value).

**Figure 4:**
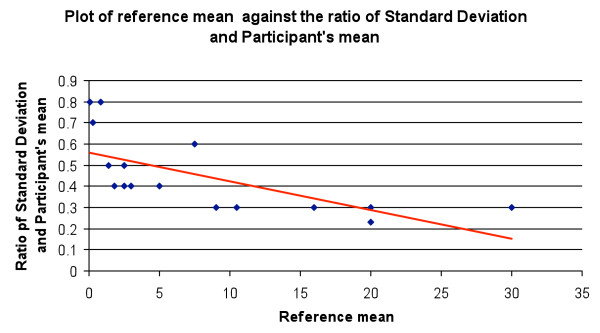
**Plot of the reference mean parasitaemia against the ratio of the standard deviation and participants' mean.** (Blue dot: ratio of SD and mean, Red line: trend line).

The higher the parasitaemia, the greater the standard deviation of the participants’ mean parasitaemia. This is demonstrated by a P value of 5.16 (regression statistics) (Figure 3), but no significant difference was found when the ratio of standard deviation and participants’ mean was compared to reference parasitaemia. (P = 0.003, regression statistics) (Figure 4).

**Figure 5:**
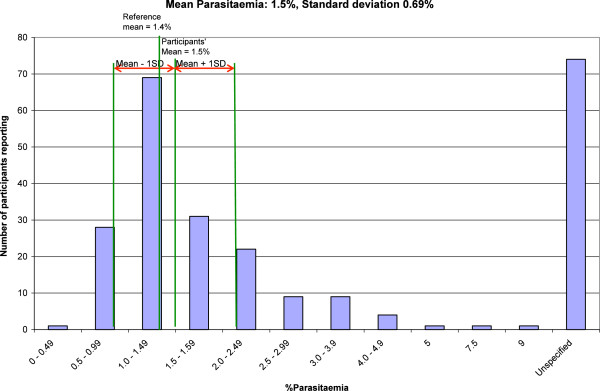
The range of parasitemia reported by participants on a blood film with an intended result of 1.5%.

**Figure 6:**
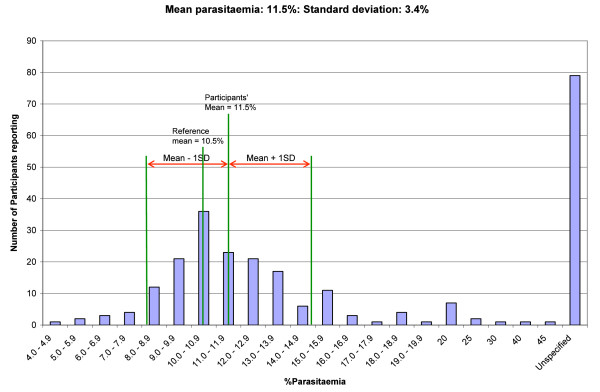
The range of parasitemia reported by participants on a blood film with an intended result of 11.4%.

**Figure 7:**
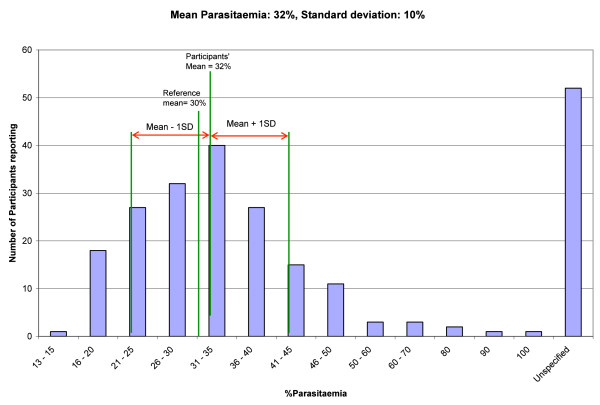
The range of parasitemia reported by participants on a blood film with an intended result of 30%.

Figures 5, 6 and 7 give graphical illustrations of the range of percentage parasitaemia reported by participants in some of the specimens containing trophozoites of *P. falciparum* distributed. They demonstrate that all specimens were prone to overestimation, under estimation (although less so with low parasitaemia) and failure to estimate the percentage parasitaemia.

Analysis of individual performance in the study showed that there was no trend suggesting that the same laboratories either overestimated, underestimated or failed to calculate the percentage parasitaemia.

## Discussion

The UK NEQAS Blood Parasitology Scheme has been distributing stained blood films for diagnosis since 1986. These specimens are designed to be assimilated into the routine workload of clinical laboratories. The reports participants receive after the close of distribution provide information allowing participants to gain an insight into their performance through the score they receive and to take individual action to investigate and remedy any incorrect results revealed when compared with the intended results obtained by the Department of Clinical Parasitology, Hospital for Tropical Diseases. The schemes are unique in that they provide relevant clinical histories with the specimen to be examined and in addition to the report of participants’ performance, a teaching sheet is issued if a poor overall performance is noted or an unusual parasite is distributed. Photomicrographs of the intended results are also featured on the UKNEQAS Microbiology website illustrating the morphological features and making recommendations for the laboratory diagnoses of the parasites that are included in distributions. These aspects emphasize the strong educational feature of the schemes.

Although the analyses of results achieved by participants in UKNEQAS Blood Parasitology Scheme for the detection and identification of blood parasites in stained thick and thin blood films in general has shown an overall improvement [5], performance data for estimation of the percentage parasitaemia of *P. falciparum* have remained unchanged. The reasons for this could be:

• Many participants fail to specify the percentage parasitaemia perhaps because it is not part of their routine practice. Counting of red blood cells infected with parasites of *P. falciparum* is essential and the percentage parasitaemia should always be reported as this has implications for prognosis and the pattern of treatment employed [6]. Participants are penalized for failing to do so.

• Overestimation of parasitaemia could to be due to their counting the number of trophozoites per 100 red blood cells and not the number of parasitized red blood cells. A red blood cell infected with multiple parasites counts as one parasitized red cell. Another reason could be including gametocytes when calculating the parasitaemia and counting all the malaria parasites present in a mixed infection could also contribute to overestimating the percentage parasitaemia. This is demonstrated in specimen 14 which contained trophozoites of *P. falciparum* with a reference parasitaemia of 16% but also contained trophozoites of *Plasmodium vivax.* 35% of participants overestimated the parasitaemia compared with 33% and 25% who overestimated the parasitaemia in specimens 15 and 16, which had a reference parasitaemia of 20%, but contained only trophozoites of *P. falciparum*. Similarly for specimen 8 which contained trophozoites of *P. falciparum* with a reference parasitaemia of 2.5%, but also contained trophozoites of *Plasmodium ovale.* Nineteen percent of participants overestimated the parasitaemia compared to 11% and 14% in specimens 6 and 7 respectively both of which had a similar parasitaemia, but contained trophozoites of *P. falciparum* alone. When calculating the parasitaemia of *Plasmodium falciparum*, only the trophozoite stages are counted and the gametocytes and other malaria parasite species are excluded from the result [6].

• Those participants who underestimate the parasitaemia may not be counting an adequate number of fields. It is recommended that 40 fields of a thin film are counted, especially when the parasitaemia is low due to the possible uneven distribution of parasites,

• Examining the wrong part of the film can contribute to an inaccurate calculation, i.e. examining fields that are too thick or too thin. The area where the red cells are touching and not overlapping or too far apart should be examined

• Calculation error is another possible source of wrong results. The recommended procedure for estimating the percentage parasitaemia in a thin blood film is by expressing the number of parasitised red blood cells per 100 cells. A minimum of 1,000 cells should be counted. The quantification can be facilitated by the use of a Miller square [6].

The reasons for misidentifying the malaria parasites present could be due to confusing Maurer’s Clefts present in the red blood cells containing trophozoites of *P. falciparum* in specimens 1, 2, 5 ,7 and 8 (Table 2) with Schüffner’s dots or James’ dots. Participants may also have confused mature trophozoites of *P. falciparum* in specimens 2 and 10 (Table 2), with those of *Plasmodium malariae*.

Bowers *et al.*[7] showed differences in the results obtained by experienced malaria microscopists using different methods to calculate the parasitaemia of *P. falciparum.* This was predominantly method rather than observer-related but it might be expected that inter-observer variation would be greater with non-specialists.

Estimation of the percentage parasitaemia in peripheral blood is essential as *P. falciparum* infection may cause fatal illness and hyperparasitaemia is a criterion for the World Health Organization’s definition of severe malaria [8]. A fall in parasitaemia also gives an indication of treatment success so failing to estimate it hampers clinical management.

UKNEQAS Parasitology continues to emphasize the importance of estimating the parasitaemia of *P. falciparum* by outlining in all the reports the consequences of not doing so in a clinical situation.

## Conclusion

The standard of parasitaemia estimations for *P. falciparum* in the UK NEQAS Blood Parasitology Scheme has not changed over 15 years largely because many participants still fail to estimate it, whilst others miscalculate.

## Competing interests

The authors declare that they have no competing interests.

## Authors’ contributions

CO collated the distribution performance data. MM drafted the manuscript and extracted the relevant data from the performance data. NA performed the statistical analyses and advised on the relevance of the statistical analyses. PLC participated in the design and coordination of the study. PLC is supported by the UCL Hospitals Comprehensive Biomedical Research Centre Infection Theme. All authors read and approved the final manuscript.
